# Case report: simultaneous occurrence of multiple myeloma and non-Hodgkin lymphoma treated by CAR T therapy

**DOI:** 10.1097/MD.0000000000019739

**Published:** 2020-04-17

**Authors:** Tongjuan Li, Jiaqi Tan, Liting Chen, Dong Kuang, Xia Mao, Yaoyao Lou, Jianfeng Zhou, Xiaoxi Zhou

**Affiliations:** a Department of Hematology; b Department of Pediatrics; c Department of Pathology, Tongji Hospital, Tongji Medical College, Huazhong University of Science and Technology, Wuhan, Hubei, China.

**Keywords:** B cell lymphoma, B cell maturation antigen, chimeric antigen receptor T, CD19, multiple myeloma

## Abstract

**Rationale:**

B cell lymphoma can co-occur with multiple myeloma (MM), and the prognosis in this case is usually poor. We propose the combination of CD19-chimeric antigen receptor (CAR) T cells and BCMA-CAR T cells for the treatment of such patients to obtain a superior prognosis.

**Patient concerns:**

We present a 50-year-old patient with previous B cell lymphoma and subsequent multiple myeloma (MM).

**Diagnosis:**

A diagnosis of B cell lymphoma and MM was made.

**Interventions:**

The patient was treated with a combination of haploidentical CD19-chimeric antigen receptor (CAR) T cells and BCMA-CAR T cells.

**Outcomes:**

After CAR T cell therapy, the monoclonal plasma cells in the bone marrow and M protein disappeared.

**Lessons:**

The combination therapy of CD19- and BCMA-CAR T cells is an effective measure to treat patients with concomitant or borderline cases of B cell lymphoma and MM.

## Abstract 2

Supplemental Digital Content is available in the text

## 1 Introduction

B cell lymphoma can be concomitant with multiple myeloma (MM), although it is not common. Of 4165 patients reported to have B cell lymphoma, 6 patients developed MM, and one of 804 patients with MM developed B cell lymphoma.^[1]^ There is no standard therapeutic regimen for such patients, and the prognosis in this case is usually poor.

Chimeric antigen receptor (CAR) T cell therapy was originally created in the 1980s, and it has been rapidly developed and has achieved inspiring outcomes in patients with B cell and plasma cell malignancies.^[2]^

There have been several well-known clinical trials of CD19 CAR T cell therapy used in recurrent/refractory (R/R) B cell lymphoma. The complete remission (CR) rate ranged from 40% to 54%, the overall response (OR) rate ranged from 52% to 82%, and the median overall survival (OS) ranged from 12 months to 18 months.^[3]^ Clinical trials of BCMA-CAR T cell therapy used in R/R multiple myeloma have also been reported. The CR rate ranged from 45% to 74%, the OR rate ranged from 81% to 94%, and the median event-free survival ranged from 31 weeks to 15 months.^[4–7]^

In this article, we report a patient with B cell lymphoma that was subsequently diagnosed with MM during disease progression who was treated with CD19-CAR T cell and BCMA-CAR T cell therapy, and her disease was effectively controlled.

## 2 Case report

**Figure 1:**
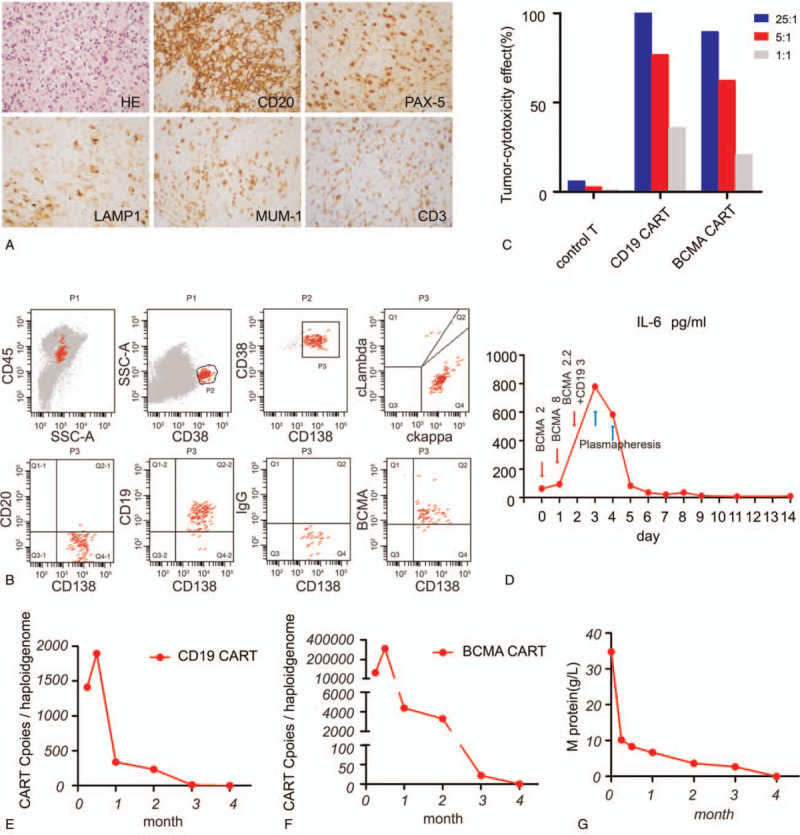
Diagnosis of2 diseases and the effect of haploidentical CAR T therapy. (A) Pathology staining of HE and some indicators such as CD20, PAX-5, LAMP1, MUM-1, and CD3 demonstrating the diagnosis of DLBCL in 2011. Photographic images were acquired with a Nikon Eclipse 50i microscope and the original magnifications were all 400x/0.95 NA. (B) The flow cytometry of bone marrow showed clonal plasma cells with abnormal expression of surface markers. (C) In vitro tumor-cytotoxicity effect in haploidentical CD19 and BCMA CAR T cells compared with control T cells at an effector/target ratio of 25:1, 5:1, and 1:1 respectively were showed. (D) IL-6 level during haploidentical CAR T therapy in first 14 days accompanied by the infusion of haploidentical CAR T cells were showed in red and the treatment of severe cytokine release syndrome by plasmapheresis was showed in blue. The first infusion day of CAR T cells was as day 0. (E) Cellular kinetics of Lentivirus’ copies of CD19 in peripheral blood after haploidentical CAR T therapy were determined by droplet digital polymerase chain reaction (ddPCR) in different time point. (F) Cellular kinetics of Lentivirus’ copies of BCMA in peripheral blood after haploidentical CAR T therapy were determined by droplet digital polymerase chain reaction (ddPCR) in different time point. (G) Immunoelectrophoresis showed the change of M protein about 4 months after haploidentical CAR T therapy.

A 50-year-old woman was diagnosed with stage I (according to Ann Arbor staging classification) MALT lymphoma (according to 2008 World Health Organization classification) by biopsy of the left parotid gland in 2009. She received 2 cycles of FC (fludarabine and cyclophosphamide (CTX)) chemotherapy and was assessed as reaching complete remission (CR). In 2011, she had lumbar and lower limb pain and was diagnosed with diffuse large B cell lymphoma (DLBCL) at Ann Arbor stage IV by vertebral biopsy (CD20+, CD30+, CD3-, PAX5+, OCT-2+, BOB.1+, CD10-, BCL6+, MUM1+, ALK−, LMP1+) (Fig. 1A) according to 2008 World Health Organization classification.^[8]^ Her bone marrow was free of tumor cells, while small numbers of IgG kappa and IgM lambda type M proteins were found by serum immunoelectrophoresis. The patient received 8 cycles of R-CHOP (rituximab, CTX, epirubicin, vindesine and dexamethasone (DXM)) chemotherapy and achieved a status of complete remission unconfirmed (CRu).

However, in 2016, by bone marrow cytomorphologic examination, 23% immature plasma cells together with 26% lymphoma cells were found. The cells were further confirmed as monoclonal cells by flow cytometry (0.2% monoclonal B cells cells: CD20+, CD22+, Kappa+, intracellular Kappa+, intracellular CD79+, CD38−; and 2.3% monoclonal plasma cells: CD19+, CD38+, intracellular Kappa+). IgG kappa, IgM lambda, and IgA kappa type M proteins were detected with serum immunoelectrophoresis, indicating 3 plasma cell tumor clones. The patient was diagnosed with concomitant multiple myeloma at R-ISS stage II and DLBCL at Ann Arbor stage IV.^[9]^ Then, the patient received 3 cycles of VD (bortezomib and DXM) chemotherapy and was assessed as reaching a partial remission (PR). After that, the patient underwent different chemotherapy regimens, including RD (lenalidomide and DXM), RVD (lenalidomide, bortezomib and DXM) and MPT (melphalan, prednisone and thalidomide). Starting December 20, 2017, the patient had intermittent fever, with a maximum body temperature of 39.6°C. Anti-infection treatment was ineffective, and the patient experienced fatigue and progressive aggravation, and the disease progressed. A brief summary of the patient's disease before CAR T therapy is listed in Supplementary Table 1. In April 2018, the patient was admitted to our hospital for further therapy.

When she came to our hospital, the patient was in poor general health and had been dealing with an intermittent fever for 4 months. Only one peak of IgG kappa type M protein (34.78 g/L) was observed by serum immunoelectrophoresis. FDG-PET/CT (positron emission tomography-computed tomography) showed metabolic increases in multiple regions: mediastinal lymph nodes, retroperitoneum, mesentery, and hepatic portal area lymph nodes. The SUVmax (standardized uptake value max) was 7.3, and the size of the largest lesion was 1.3 × 1.1 cm. A total of 0.27% monoclonal plasma cells in the bone marrow expressing CD38, CD138, CD19, intracellular kappa and BCMA were detected by flow cytometry (Fig. 1B). The results of FISH (fluorescence in situ hybridization) and NGS (next-generation sequencing) were negative.

Considering the history of lymphoma, we decided to employ CD19-CAR T cells combined with BCMA-CAR T cells for further treatment. There is a clinical trial of anti-CD19 chimeric antigen receptor-modified T cell (anti-CD19 CAR T cell) therapy for relapsed, refractory and high-risk CD19+ B cell malignancies (ClinicalTrials.gov number ChiCTR-OIN-16007723), and there is an open single-center, single-arm clinical study for anti-BCMA CAR T cell therapy for relapsed, refractory and high-risk BCMA+ tumors (ClinicalTrials.gov number ChiCTR-OPC-16009113). Unfortunately, T lymphocytes from the patient did not expand in vitro and thus could not be used for the preparation of CAR T cells. Therefore, T lymphocytes from her son were used alternatively, although the patient had not previously received transplantation of allogeneic hematopoietic stem cells. The haplo-CAR T cells proliferated in vitro very well. As shown in Figure 1C, the tumor-cytotoxic rates of CD19-CAR T cells and BCMA-CAR T cells were 99.93% and 89.28% at an effector/target ratio of 25:1, with 21.2% and 37.8% infection efficiencies, respectively. After 3 days of standard FC (fludarabine 25 mg/m^2^ and cyclophosphamide 20 mg/kg) lympho-depleting chemotherapy, day 0 was defined as the first day of infusion. BCMA-CAR T cells (1.22 × 10^7^ cells/kg) and CD19-CAR T cells (3 × 10^6^ cells/kg) were infused from day 0 to day 2 (Fig. 1D). The patient had a high fever that lasted 26 hours with 5 fever peaks; the highest temperature reached 39.6°C, and the cytokine release syndrome grade was 2. At day 3 after infusion, the serum level of IL-6 was elevated to 779.7 pg/mL, which was increased by 12.2-fold compared with the baseline concentration of IL-6 on day 0. Plasmapheresis was used twice on days 3 and 4 (Fig. 1D). Meanwhile, the lentivirus copy numbers of CD19-CARs and BCMA-CARs reached their peaks at 1410 copies/μg and 311,561 copies/μg, respectively, on day 14 (Fig. 1E). After CAR T cell therapy, the temperature returned to normal, and her fatigue was relieved gradually. Although the lentivirus copy numbers were very low at the third month after CAR T therapy, reexaminations of serum immunoelectrophoresis showed a decrease in M protein, and M protein disappeared at 4 months, as shown in Figure 1F. In the bone marrow, both monoclonal B lymphoma cells and monoclonal plasma cells were also undetectable. We speculate that the tumor cells might have been cleared at the third month, while because of the long half-life of the M protein, the M protein did not disappear until the fourth month after CAR T therapy. Unfortunately, PET-CT reexamination was not conducted because of patient financial reasons.

The details of the methods are shown in the supplementary materials.

## 3 Discussion

The patient we presented was originally diagnosed with lymphoma. However, in 2016, both monoclonal plasma cells and monoclonal B cells were found in her bone marrow, and multiple IgG kappa, IgM lambda, and IgA kappa monoclonal proteins were detectable by serum electrophoresis, indicating multiple plasma cell tumor clones. Specifically, the patient suffered from both lymphoma and multiple myeloma. The sequential changes of the patient seen on biopsy indicated different stages during the development of the disease. The pathological mechanism may involve lymphoma cell differentiation into plasma cells under therapeutic pressure, eventually causing the development of plasmacytoma. Because there is no molecular evidence to identify whether the MM developed from lymphoma, it is also possible that the 2 tumors occurred independently at the same time or subsequently.

In addition, we reviewed 8 similar cases from the PubMed database. The features, treatment and progress of the cases are listed in Table 1. Among the cases, there were 6 cases with concomitant B cell lymphoma and multiple myeloma and 2 borderline cases with pathological and histological features of both B cell lymphoma and multiple myeloma.^[10–17]^ The treatment of B cell lymphoma is mainly composed of immunotherapy with a CD20 monoclonal antibody together with chemotherapy, while the treatment of MM is mainly composed of proteasome inhibitors and chemotherapy. Because of the lack of standard treatment guidelines for both diseases, different chemotherapy regimens were used for these patients. Among the 6 patients, 2 patients were lost to follow-up after CVP (cyclophosphamide 750 mg/m^2^, vindesine 4 mg, and prednisone 100 mg/m^2^) chemotherapy or 2 cycles of R-CHOP (rituximab 375 mg/m^2^ (d0), CTX 750 mg/m^2^ (d1), epirubicin 90 mg/m^2^, (d1), vindesine 4 mg (d1), and dexamethasone (DXM) 15 mg (d1–5)); another 2 patients relapsed one or more times. Of these 2 patients, one patient underwent 6 cycles of COPP/ABVD together with radiotherapy, 1 cycle of ChIVPP, 2 cycles of melphalan and 3 cycles of ABVD. The other patient underwent 6 cycles of DCEP and 1 cycle of ECHOP (Some chemotherapy regimens did not give specific dosages and usage). One patient received chemotherapy and maintained a CR for the lymphoma and a stable disease (SD) state for the MM within 2 years of follow-up. The last patient had both IgM kappa and IgA kappa monoclonal proteins in serum, and after therapy, the IgA kappa monoclonal protein disappeared, while the IgM kappa monoclonal protein remained constant. The 2 borderline patients failed to respond to chemotherapy and died because of disease development. The characteristics of B cell lymphoma and multiple myeloma are different, which is probably the reason for these patients’ poor prognosis. We need a regimen that can treat these two diseases simultaneously with acceptable toxicity and side effects.

**Table 1:**
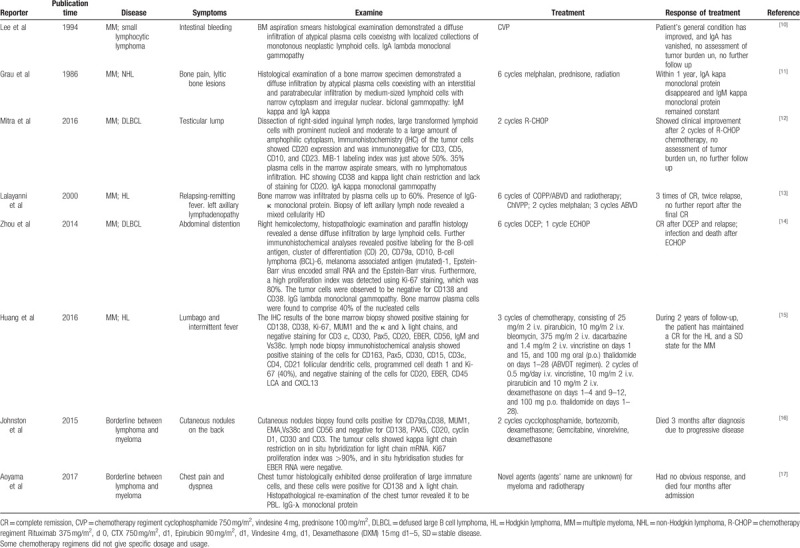
Cases or borderline cases of coexistence of MM and B-cell lymphoma.

Our patient had both B cell lymphoma and MM. When she came to our hospital, there were 0.27% monoclonal plasma cells in the bone marrow expressing CD19 and BCMA. Therefore, we chose CD19- and BCMA-CAR T cell therapies. In addition, CD19-CAR T cell therapy is also effective in controlling B cell lymphoma, although the patient had no lymphoma cells in the bone marrow. However, we could not absolutely exclude this disease because of her medical history. As her own T cells failed to proliferate in vitro, she could only receive haplo-identical CAR T cell therapy, and her disease was controlled very well. The amplification of the haplo-identical CAR T cells was very good in vivo, and there was no graft-versus-host disease. Thus, the combination therapy of CD19- and BCMA-CAR T cells is an effective measure to treat concomitant or borderline cases of B cell lymphoma and MM. Moreover, if the patient has not received transplantation and the viability of the patient's T cells is low, induction of proliferation or transduction of a CAR is difficult, the tumor-cytotoxic effect of the T cells is poor in vitro, or there is potential T cell immunodeficiency, haplo-identical CAR T therapy is also an option.

In summary, the combination therapy of CD19- and BCMA-CAR T cells is an effective measure to treat concomitant or borderline cases of B cell lymphoma and MM. For patients who have not received transplantation with low T cell viability, haplo-identical CAR T therapy is also an option.

## Acknowledgments

The authors would like to thank all members of the study team, the patient, and their family. We would also thank the wonderful work of Bio-Raid Company, especially in the preparation of CAR T cells.

## Author contributions

Xiaoxi Zhou analyzed and interpreted the data; Xia Mao conducted the experiments of flow cytometry; Dong Kuang analyzed pathological section; Liting Chen and Yaoyao Lou evaluated the lentivirus copy numbers; Tongjuan Li and Jiaqi Tan managed patient and wrote the manuscript. All authors read and approved the final manuscript; Jianfeng Zhou participated in reviewing of the article.

**Conceptualization:** Xiaoxi Zhou.

**Funding acquisition:** Tongjuan Li, Xiaoxi Zhou.

**Investigation:** Tongjuan Li, Liting Chen, Dong Kuang, Xia Mao, Yaoyao Lou.

**Methodology:** Liting Chen, Dong Kuang, Xia Mao, Yaoyao Lou.

**Writing – original draft:** Tongjuan Li, Jiaqi Tan.

**Writing – review & editing:** Jianfeng Zhou.

## Supplementary Material

Supplemental Digital Content

## Supplementary Material

Supplemental Digital Content
