# Supplementary material for: Case report: simultaneous occurrence of multiple myeloma and non-Hodgkin lymphoma treated by CAR T therapy
Source: Medicine (Baltimore). 2020 Apr 17;99(16):e19739. doi: 10.1097/MD.0000000000019739 (PMC7220358; doi:10.1097/MD.0000000000019739)
Supplement: Supplemental Digital Content [file medi-99-e19739-s001.docx]

**Supplementary Table 1. Disease diagnosis and treatment**

| Time of diagnosis | Diagnosis, stage | Biopsy and immunohistochemistry | Bone marrow involvement | Imageological Examination | M protein type | Treatment and efficacy | Adverse Events |
| --- | --- | --- | --- | --- | --- | --- | --- |
| 2009/4/10 | MALT^a^, I | left parotid gland^b^ | negetive | ultrasonography^c^ | none | FC×2(CR) ^d^ | No data* |
| 2011/11/16 | DLBCL^e^, IV | T12 ,L5^f^ | negetive | PET/CT^g^ | IgG kappa, IgM lambda | R-CHOP×8(Cru)^h^ | No data* |
| 2016/3/30 | MM^i^, II | none | monoclonal plasma cells^j^ | PET/CT^l^ | IgG kapa(65.6g/L), IgM lambda(2.32g/L), IgA kappa(18.6g/L) | VD×3(PR), RD, RVD, MPT (PD)^m^； CAR T therapy (CR)^n^ | numbness in hands, pain in feet, grade2 neurotoxicity； grade 2 cytokine releasing syndrome of CAR T therapy |
|  | DLBCL, IV |  | monocloonal B cells^k^ |  |  |  |  |

1. MALT: mucosa-associated lymphatic tissue.
2. CD20+, CD15-, CD30-, CD3+, CD43+, CD79a+, EMA-, Ki67+, BCL2-;
3. Ultrasonography showed a 2.5×1.3 cm nodule on the left side of the face;
4. FC: fludarabine and cyclophosphamide; CR: complete remission;
5. DLBCL: diffuse large B cell lymphoma;
6. T: thoracic vertebra; L: represents lumbar vertebra; CD20+, CD30+, CD3-, PAX5+, OCT-2+, BOB.1+, CD10-, BCL6+, MUM1+, ALK-, LMP1+;
7. PET/CT showed metabolic increasement in multiple regions: diaphragm angle, retroperitoneum, T1 T12 L1 L5 vertebral body, left mandible, left superior humerus, left clavicular acromion, sternal stalk, bilateral ilium, right side of sacrum, and right side of femur trochanter;
8. R-CHOP, rituximab, CTX, epirubicin, vindesine and dexamethasone (DXM); CRu, complete remission unconfirmed;
9. MM: multiple myeloma;
10. 23% immature plasma cells by bone marrow cytomorphologic examination; and 2.3% monoclonal plasma cells (CD19+, CD38+, intracellular Kappa+) by flow cytometry;
11. 26% lymphoma cells by bone marrow cytomorphologic examination; and 0.2% monoclonal B cells cells (CD20+, CD22+, Kappa+) by flow cytometry;
12. PET/CT showed metabolic increasement in multiple regions: right costal angle, hilar area, para-pancreatic head, retroperitoneum and double inguinal;
13. VD: bortezomib and dexamethasone; PR: partial remission; RD, lenalidomide and dexamethasone; RVD, lenalidomide, bortezomib and dexamethasone; MPT, melphalan, prednisone and thalidomide; PD, progressed disease;
14. CAR: chimeric antigen receptor;

*) Details are unknown because the patient was treated in other hospital.
