# Supplementary material for: Case report: simultaneous occurrence of multiple myeloma and non-Hodgkin lymphoma treated by CAR T therapy
Source: Medicine (Baltimore). 2020 Apr 17;99(16):e19739. doi: 10.1097/MD.0000000000019739 (PMC7220358; doi:10.1097/MD.0000000000019739)
Supplement: Supplemental Digital Content [file medi-99-e19739-s002.docx]

**Materials and Methods**

**1. Ethics approval and consent to participate**

This study was approved by the Medical ethics committee of Department of Hematology, Tongji Hospital, Tongji Medical College, Huazhong University of Science and Technology (TJ-IRB20160315, TJ-IRB20160312). The patient and donor gave their written informed consent in accordance with the Declaration of Helsinki. This study is registered at www.chictr.org.cn as ChiCTR-OIN-16007723 and ChiCTR-OPC-16009113.

**2. Generation of CAR-T cells**

Lymphocytes were obtained from the patient or her son by apheresis and the CD3-positive T cells were isolated by magnetic bead-conjugated anti-CD3 antibody (MiltenyiBiotec 130-050-101). Then the enriched CD3-positive T cells were stimulated with anti-CD3/anti-CD28 monoclonal antibody-coated magnetic beads（Thermo Fisher 11132D）and cultured for 2 days in modified CTS™ OpTmizer™ T-Cell Expansion SFM (Thermo Fisher A10221-01) at 37 °C, 5% CO_2_. The cells were transduced with lentiviral vector encoding anti-CD19 single-chain variable fragment, linked with CD28 and 4-1BB costimulatory domains and CD3-ζ signaling domain, or anti-BCMA single-chain variable fragment, linked with CD28 and CD3-ζ signaling domains (multiplicity of infection MOI = 3), respectively, and cultured for 10-14 days. During culture, the medium was renewed every 3 days and the cells were controlled to maintain a concentration of 1.5-2.0×10^6 /ml. Only when the transduction efficiencies and the tumor cytotoxic effects of CAR T-cells were optimal, the T cells could be infused into the patient.

**3. Cellular kinetics.**

CD19 or BCMA CAR gene specific minor groove binder (MGB) probe was designed by Primer Express 3.0 respectively. The absolute quantification of CAR gene copy numbers was determined by droplet digital polymerase chain reaction (ddPCR) before or after infusion of CAR T cells. The primers including CD19_forward_: CCGGCTGACCATCATCAAG; CD19_Reverse_: GGTCTGCAGGCTGTTCATCTT; CD19_Prob_: CAACAGCAAGAGCCAGG; BCMA_forward_: AAGTGGATGGGCTGGATCAA; BCMA_Reverse_: TCTGCCCCGGAAGTCGTA; BCMA_Prob_: ACCGAGACAAGAGAGC, were synthesized by Invitrogen Company (Shanghai, China). We used noncompartmental methods to assess the cellular kinetic parameters (CD19 and BCMA) (AUC0-28d, Cmax, Tmax, Tlast, T1/2) by NonCompart package of R language.

**4. Evaluation of cytotoxic effect of CAR T cells in vitro**

Target cells, including U266 for BCMA-CAR T cells and Raji for CD19-CAR T cells, were incubated with 25 μM calcein-AM at 37°C for 30 min in the presence of 5% FBS. After washing, target cells were mixed with CAR T cells and control T cells at E/T (effector and target cells) ratio of 25:1, 5:1 and 1:1. Target cells alone served as a negative control, and target cells in lysis buffer served as a positive control. After incubation for 2 h, the mixture was centrifuged, and the supernatant was obtained for evaluation of calcein-AM. The cytolytic effect was calculated by the following formula: Cytotoxicity (%) = (OD experimental group – OD negative control) / (OD positive control – OD negative control) ×100.
